# Spinal Deformity in Aged Zebrafish Is Accompanied by Degenerative Changes to Their Vertebrae that Resemble Osteoarthritis

**DOI:** 10.1371/journal.pone.0075787

**Published:** 2013-09-24

**Authors:** Anthony J. Hayes, Scott Reynolds, Mari A. Nowell, Lee B. Meakin, Judith Habicher, Johan Ledin, Andrew Bashford, Bruce Caterson, Chrissy L. Hammond

**Affiliations:** 1 Pathophysiology and Repair, Cardiff School of Biosciences, Cardiff University, Cardiff, Wales, United Kingdom; 2 Departments of Biochemistry and Physiology & Pharmacology, University of Bristol, Bristol, England, United Kingdom; 3 Inflammation, Skin & Joint Disease, Institute of Infection & Immunity, School of Medicine, Cardiff University, Cardiff, Wales, United Kingdom; 4 School of Veterinary Science, University of Bristol, Bristol, England, United Kingdom; 5 Evolutionary Biology Centre, Uppsala University, Uppsala, Sweden; Faculté de médecine de Nantes, France

## Abstract

Age-related degenerative changes within the vertebral column are a significant cause of morbidity with considerable socio-economic impact worldwide. An improved understanding of these changes through the development of experimental models may lead to improvements in existing clinical treatment options. The zebrafish is a well-established model for the study of skeletogenesis with significant potential in gerontological research. With advancing age, zebrafish frequently develop gross deformities of their vertebral column, previously ascribed to reduced trunk muscle tone. In this study, we assess degenerative changes specifically within the bone and cartilage of the vertebral column of zebrafish at 1, 2 and 3-years of age. We show increased frequency and severity of spinal deformities/curvatures with age. Underlying the most severe phenotypes are partial or complete vertebral dislocations and focal thickening of the vertebral bone at the joint margins. MicroCT examination demonstrates small defects, fractures and morphological evidence suggestive of bone erosion and remodeling (i.e. osteophytes) within the vertebrae during aging, but no significant change in bone density. Light and electron microscopic examination reveal striking age-related changes in cell morphology, suggestive of chondroptosis, and tissue remodelling of the vertebral cartilage, particularly within the pericellular micro-environment. Glycosaminoglycan analysis of the vertebral column by HPLC demonstrates a consistent, age-related increase in the yield of total chondroitin sulfate disaccharide, but no change in sulfation pattern, supported by immunohistochemical analysis. Immunohistochemistry strongly identifies all three chondroitin/dermatan sulphate isoforms (C-0-S, C-4-S/DS and C-6-S) within the vertebral cartilage, particularly within the pericellular micro-environment. In contrast, keratan sulfate immunolocalises specifically with the notochordal tissue of the intervertebral disc, and its labelling diminishes with age. In summary, these observations raise the prospect that zebrafish, in addition to modelling skeletal development, may have utility in modelling age-related degenerative changes that affect the skeleton during senescence.

## Introduction

The vertebral column, ‘backbone’ or spine, is the central defining feature of vertebrates. It consists of a series of interconnected vertebrae, separated by flexible intervertebral discs (IVDs) that span the dorso-medial aspect of the organism. As the vertebral column ages, it undergoes progressive and irreversible degenerative changes that can lead to back pain, deformity and disability [1,2]. Age-related degenerative changes within the spine are shaped by genetics, social environmental and occupational factors and can affect diverse spinal tissues; however, those most affected are generally the vertebrae and the IVDs [1,3,4,5,6].

During aging, pathological changes in the extracellular matrix (ECM) of the IVD can lead to joint space narrowing, joint instability and nerve impingement [7]. This can result in inflammation, pain and tissue remodeling leading to calcification or ossification of the disc, and the formation of bony spurs, or osteophytes, on the lateral margins of vertebral bodies (VBs) [8]. [9]. Progressive loss of bone mass (osteoporosis) within the vertebral bodies and osteoarthritic changes (e.g. erosion of articular cartilage and osteophytosis) in the facet joints at the back of the vertebral column contribute to these changes and affect associated spinal connective tissues [9,10,11]. The cumulative effect of these age-related pathologies is pain, deformity and morbidity.

Fish are well-suited to studies of spinal integrity without the biomechanical constraints observed in terrestrial vertebrates [12]. The zebrafish (*Danio rerio*), in particular, is well-established as a model for the study of skeletogenesis, showing many parallels with higher vertebrates in the development of its vertebral column [13,14,15,16,17,18]. Zebrafish have also been proposed as a model to study vertebral body fusion [19], scoliosis [20] and as a gerontological model for studies into human aging research [21,22]. Interestingly, during aging, zebrafish naturally develop spinal curvatures which, it has been postulated, result from sarcopenia or atrophy of spinal muscles [23].

Whilst zebrafish have been shown to have validity as a model in osteoarthritis research: homologous zebrafish genes have been identified for six of the major osteoarthritis susceptibility genes [24], it is unclear whether this organism develops degenerative joint disease with age. In this study, we hypothesize that spinal curvature in fish is underpinned by age-related degenerative changes within the cartilage and bone of the vertebral column, as occurs in many human arthropathies. Our data indicates that these tissues undergo significant age-related changes in their macromolecular structure, organisation and composition, which may cause, or contribute to, the spinal deformities seen in old fish. These observations indicate that zebrafish, in addition to modelling skeletal development, has validity in modelling degenerative changes that affect the skeleton with age. 

## Materials and Methods

### Husbandry and processing of fish

Zebrafish (*Danio rerio*), raised as previously described [25], were fixed (see below) at 1, 2 and 3-years of age. Fish were morphologically evaluated by subjective visualisation into 4 categories (suspected dislocation, severe dislocation, mild curvature or no deformity) and photographed in sagittal and coronal orientations. A minimum of 3 fish were examined at each developmental stage for each experiment (see table 1). All procedures were cleared by the University of Bristol Ethical Review Committee and were performed under Home Office Project Licence number 30/2863.

**Table 1:** Summary of samples used for experimental analyses.

**Procedure**	**Sample size**	
Gross morphology	20 fish per age group	
Radiography	4 x 1 year fish, 3 x 2 year fish, 5 x 3 year fish	Low power imaging of whole spines; high power imaging of cervical and trunk regions
MicroCT	3 fish per age group for each separate analysis	3 replicates (cervical vertebrae)
Histology		Serial sagittal sections (whole spines)
Electron Microscopy		Ultrathin sections at 3 different depths (cervical vertebrae)
Immunohistochemistry		3 replicates (mid-sagittal sections of whole spines)
RPIP-HPLC		3 replicates (whole spines)

RPIP-HPLC, Reverse Phase Ion Pair-High performance liquid chromatography.

### Radiography

Specimens, fixed in 4% paraformaldehyde, were x-rayed in sagittal and coronal orientations. Radiographic images of the whole skeleton were captured using a KODAK In-Vivo Imaging System FX Pro (Kodak Molecular Imaging^TM^ Systems, USA) using an accelerating voltage of 35kVp and with an f-stop of 4.2 for 60 seconds. Images were acquired and analysed using KODAK Molecular Imaging^TM^ V3 software.

### MicroCT

Ethanol-fixed samples were descaled, eviscerated, and gently wrapped in PVC-free clingfilm. Vertebral topography was imaged in a SkyScan 1172 high-resolution micro-CT scanner (Bruker, Kontich, Belgium) using a 4.8µm voxel size. The applied x-ray voltage was 50kV with 0.5mm aluminum filtration. Scans were over 180° with a 0.6° rotation step. Images were reconstructed and binarised with global thresholding using SkyScan CTAn software, as described [26]. A region of interest was traced around individual vertebrae and surface-rendered models prepared using the “Double Time Cubes” 3D reconstruction method [27]. Cortical bone mineral density (BMD) was estimated by comparing bone density with calibration phantoms of known BMD, scanned at the same time as the vertebrae.

### Histology

Paraformaldehyde-fixed samples were processed into wax and sagittally-sectioned at 8µm. To selectively contrast matrix proteoglycans (PGs), sections were de-waxed and stained with 1% Alcian blue 8GX (pH 2.5) for 30 mins, followed by haematoxylin and eosin for tissue context. To reveal collagen fibre organisation, sections were stained in 0.1% Sirius red F3B in saturated aqueous Picric acid for 30 mins. Sections were washed, dehydrated and mounted under coverslips with DPX mountant. Regions of interest were then photographed on a Leica DM6000 photomicroscope (Leica, Heidelberg, Germany) using brightfield optics for visualisation of Alcian blue staining and polarising optics for Picrosirius red.

### Electron Microscopy

Spinal segments (C2-C3; C4-C5) from each age group (n=3) were fixed in cold 2.5% glutaraldehyde and 4% paraformaldehyde in 0.1M sodium cacodylate (pH 7.4) for 1 h, then post-fixed in 1% OsO4 in 0.1M sodium cacodylate for 1 h. Specimens were dehydrated, cleared and then infiltrated and embedded in Epon^TM^ resin. Ultrathin sections were taken at three different levels within the tissue, stained with 7% uranyl acetate and lead citrate and examined on a FEI Tecnai^TM^ 12 transmission electron microscope. Photomicrographs were imported into ImageJ for size quantification of chondrocyte lacunae, a minimum of 10 cells and associated lacunae were measured from each of 3 different fish at each age.

### Immunohistochemistry

To evaluate glycosaminoglycan (GAG) distribution, de-waxed spinal sections were labelled with a panel of well-characterised monoclonal antibodies (see table 2) towards chondroitin sulfate/dermatan sulfate (CS/DS) and keratan sulfate (KS) GAGs using the Vector ABC Universal Elite peroxidase kit (Vector Laboratories, U.S.A). Sections were pre-incubated with chondroitinase ABC (0.5 U/ml; Sigma-Aldrich, UK) in 0.1M Tris acetate (pH 7.5) for 1 h at 37°C to generate antibody recognition sites within the CS/DS GAG chains. Sections were then rinsed in water and incubated in 3% H_2_O_2_ in methanol for 1 h, to eliminate endogenous peroxidase activity. Sections were washed in phosphate buffered saline (pH 7.4) containing 0.01% Tween 20 (PBST) then blocked in horse serum (2.5%) for 30 mins. After a brief rinse, endogenous avidin and biotin were blocked using a commercial kit (Vector Laboratories, U.S.A). Sections were incubated overnight with primary antibody at 4°C (table 2). To test for non-specific labelling, the primary antibody was omitted or replaced with naive immunoglobulin. Sections were subsequently washed in PBST, incubated with biotinylated secondary antibody for 30mins; washed again, then incubated with avidin-biotin-complex reagent for 30mins. Peroxidase was localized using a Vector® NovaRED™ Substrate Kit (Vector Laboratories, U.S.A). Sections were counterstained with Mayer’s haematoxylin, before mounting under coverslips with DPX mountant (Sigma-Aldrich, UK). Regions of interest were photographed under brightfield optics using a Leica DM6000 microscope (Leica, Heidelberg, Germany).

**Table 2:** Antibodies used in immunohistochemistry.

**Antibody (dilution**)	**Isotype**	**Pre-treatment**	**Specificity**	**Source/ Reference**
1B5 (1:5)	IgG1,kappa	ABC	C-0-S	[62,63]
2B6 (1:5)	IgG1,kappa	ABC	C-4-S/DS	[62,63]
3B3 (1:5)	IgM, kappa	ABC	C-6-S	[63]
5D4 (1:5)	IgG1,kappa	ABC*	KS	[64]

ABC, chondroitinase ABC; C-0-S, chondroitin-0-sulfate; C-4-S, chondroitin-4-sulpfate; C-6-S, chondroitin-6-sulfate; DS, dermatan sulfate; KS, keratan sulphate; * chondroitinase ABC was used to remove CS chains for improved KS labelling.

### Reverse Phase Ion Pair (RPIP)-high performance liquid chromatography (HPLC)

CS was isolated from vertebral columns (head and fins first removed; with 3 replicates performed at each age), degraded into disaccharides by enzymatic cleavage, and detected in a HPLC-based system using published methodology [28]. Briefly, GAGs were isolated by proteolytic cleavage, nuclease treatment, and diethylaminoethyl ion exchange chromatography. The purified GAGs were cleaved with chondroitinase ABC (0.5 U/ml, Seikagaku, 40mM Tris-Ac, pH8.0) and analysed to identify CS disaccharide components. CS disaccharides were subjected to RPIP-HPLC analysis followed by post-column derivatization with 2-cyanoacetamide (0.25% in 0.5% NaOH) and detection in a fluorescence detector. The identity and the amount of the disaccharides was established by comparing the samples with CS disaccharide standards. 

## Results

### Morphology

**Figure 1:**
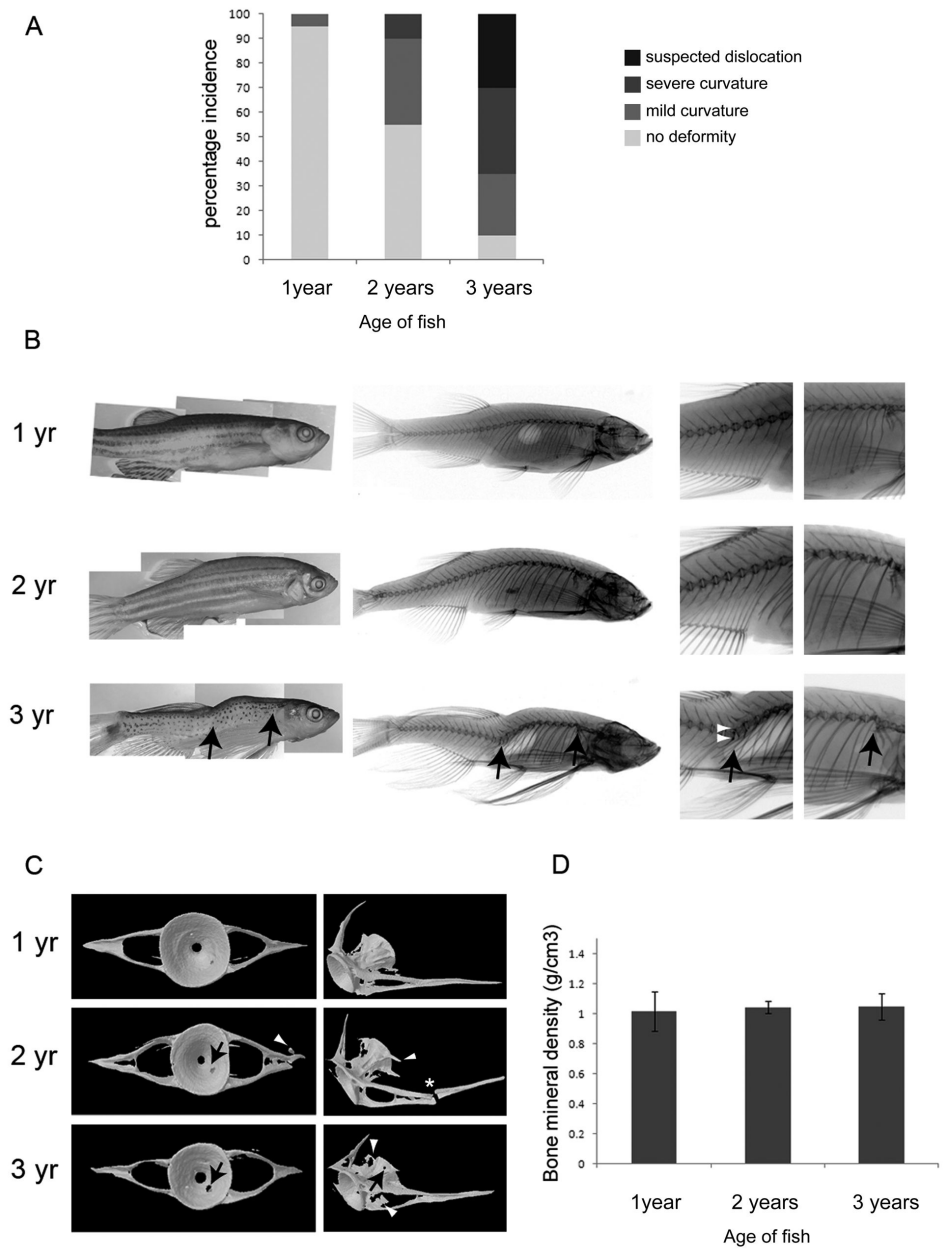
Aging zebrafish show gross morphological changes to the vertebral column. **A**. Graph showing incidence (%) of deformities by age. n=20 for each age group. **B**. Gross morphological appearance (left panel) and corresponding radiology (middle and left panels; left panels show detail of trunk and tail vertebrae) of zebrafish at 1, 2 and 3-years. Black arrows (in bottom panel) denote suspected dislocations of the spine. White arrowheads point to regions of increased bone density in vertebrae surrounding the dislocation. **C**. MicroCT images showing a representative single reconstructed vertebra (C5) from each age group, black arrows point to regions of bone erosion, white arrowheads point to bony outgrowths; *asterisk* denotes fracture. **D**. Graph of average bone mineral density shows no difference to bone density at the different ages, tested by One-way ANOVA; 1 vs 2 year *P*=0.80, 1 vs 3 year *P*=0.92, 2 vs 3 year *P*=0.79. n=3 for each age.

Gross morphological evaluation of fish indicated a progressive increase in the incidence and severity of spinal curvatures with age (Figure 1A,B), as shown previously [23]. In 1 year samples, there was a low incidence of spinal abnormalities, with the majority of the fish exhibiting a generally smooth, linear contour. At 2-years, spinal curvatures became more common. At 3-years, most of the fish displayed some form of curvature (lordosis, kyphosis or scoliosis) with the majority exhibiting severe spinal deformity suggestive of a vertebral dislocation (Figure 1B; left panel).

### Radiography

Radiographic analysis showed abrupt displacements of the vertebral column in the most severely deformed 3 year fish, that were not apparent at 1- or 2-years (Figure 1B; middle panel). These outward displacements were identifiable both within the trunk and tail and occurred in dorsal, ventral and lateral planes, reflecting the spinal curvatures that were manifest in the external appearance of the fish. Higher power radiographic observation at these sites revealed partial or total vertebral dislocation and focal thickening of the bone at the joint margins (Figure 1B; bottom right panels).

### MicroCT

Microtomographic analysis of fish vertebrae at each age revealed striking differences in their morphology (Figure 1C). In 1-year specimens, the vertebrae were roughly symmetrical in shape, with well-defined contours and a smooth surface morphology. At this age, there was little evidence of bone erosion or tissue remodeling of the vertebral body, or paired transverse processes. At 2-years, the vertebrae were less symmetrical and more misshapen in appearance. The vertebral bodies had irregular contours and there was morphological evidence suggestive of tissue remodeling, with some samples exhibiting prominent bony outgrowths at their margins, characteristic of osteophytes. By 3-years, there was widespread morphological evidence of bone erosion, pitting and tissue remodeling within the vertebrae; however, analysis of bone mineral density showed similar values (g/cm3) for vertebrae at each time point studied over the three year period, indicating no underlying osteoporotic changes (Figure 1D).

### Histology and ultrastructure

**Figure 2:**
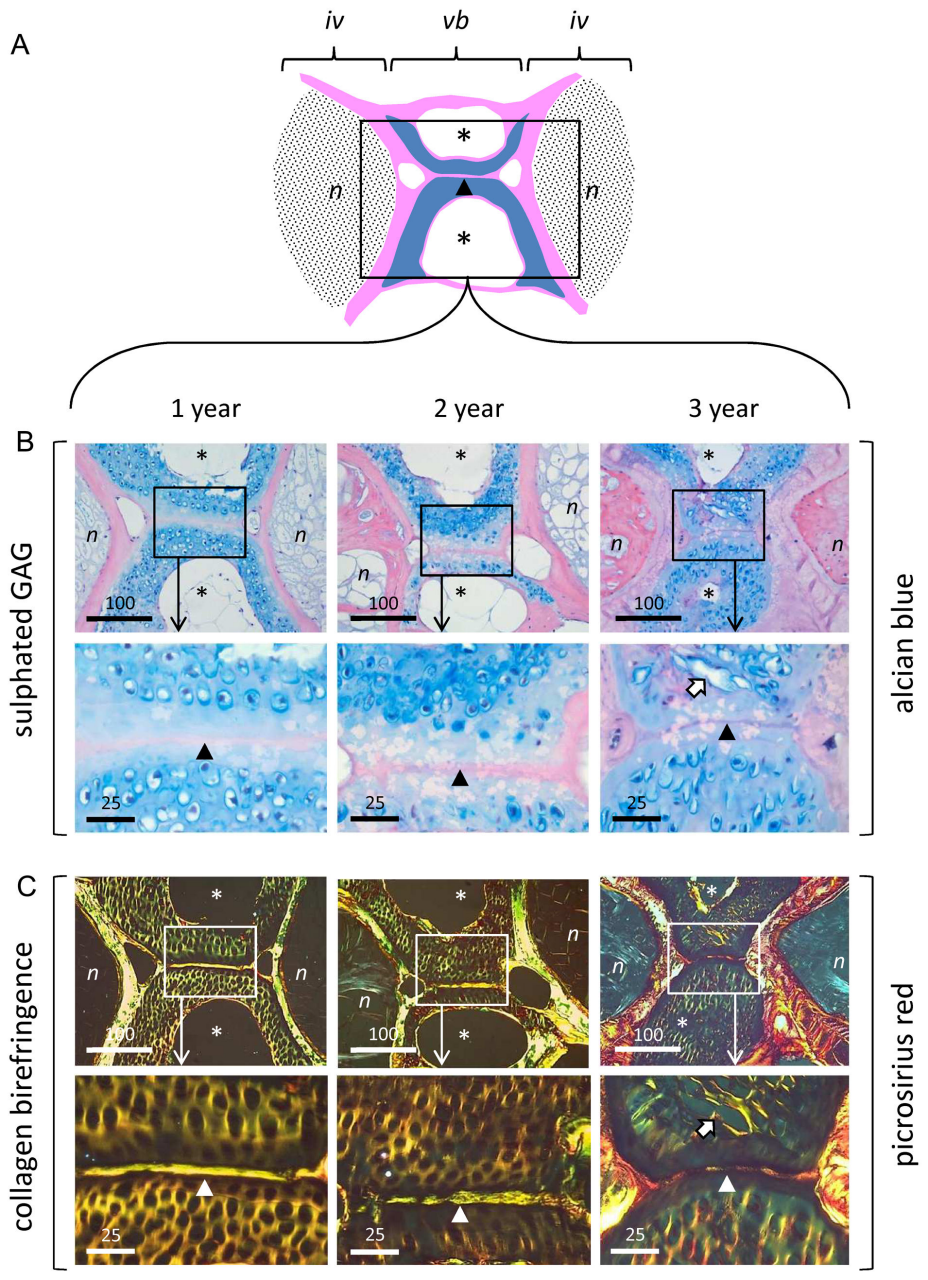
Aging zebrafish show changes in matrix organisation visible at the light microscopic level. **A**. Schematic showing anatomical organisation of trunk vertebrae/intervertebral disc (mid-sagittal section plane). Vertebral bone depicted in pink; vertebral cartilage in blue; intervertebral discs shaded in black. **B**. Alcian blue staining of sulfated GAG in 1, 2, and 3 year spines. *Upper* *panel*: low power showing discs and interjacent vertebral body. Boxed areas denote regions examined at high power in underlying panel. *Lower* *panel*: Detail of Alcian blue staining within vertebral cartilage. Note prominent pericellular localisation of GAG and change in chondron morphology with age. **C**. Collagen birefringence (Picrosirius red staining under polarising optics) in 1, 2 and 3 year spines. *Upper* *panel*: low power showing discs and interjacent vertebral body. Boxed areas denote regions examined at high power in underlying panel. *Lower* *panel*: detail of collagen birefringence within vertebral cartilage. N.B. collagen birefringence occurs throughout the ECM at 1 and 2-years, but becomes increasingly organised within the pericellular matrix by 3-years. *iv*, intervertebral disc; *vb*, vertebral body; *n*, notochord-derived tissue; *asterisks*, cavities within cortical bone of vertebrae; *arrow-head*, notochordal tract running through cartilaginous inner facet of vertebral bodies; *block* *arrow* denotes coalescence of adjacent chondrons in 3 year samples. Scalebar in microns.

Histological staining of mid-sagittal sections through fish at each age with Alcian blue identified the GAG-rich, cartilaginous inner facet of the vertebral body (Figure 2A,B). Staining was greatest within the pericellular matrix (i.e. the chondron capsule) with weaker staining of the surrounding interstitial ECM. At 1 and 2-years, chondrons were roughly spherical in morphology; however, by 3-years, they were more oval or irregular in shape, with conspicuously larger lacunae. Some lacunae were empty; others had coalesced at their margins with a loss of interstitial matrix (block arrow). The cartilage and bone had thickened considerably in 3 year vertebrae, and the intervertebral disc contained eosinophilic tissue, that appeared distinct from the vacuolated tissue of notochordal origin seen at 1 and 2-years.

Polarising microscopy of Picrosirius red-stained sections showed similar age-related features within the cartilage and bone of the vertebral column, and provided detail of underlying collagen organisation (Figure 2C). At 1 and 2-years, strong collagen birefringence was noted throughout the vertebral cartilage. In marked contrast, in 3 year samples, collagen birefringence was prominent mainly within the pericellular micro-environment. The adjacent bone was highly birefringent at all ages, particularly at 3-years, consistent with on-going collagen deposition.

**Figure 3:**
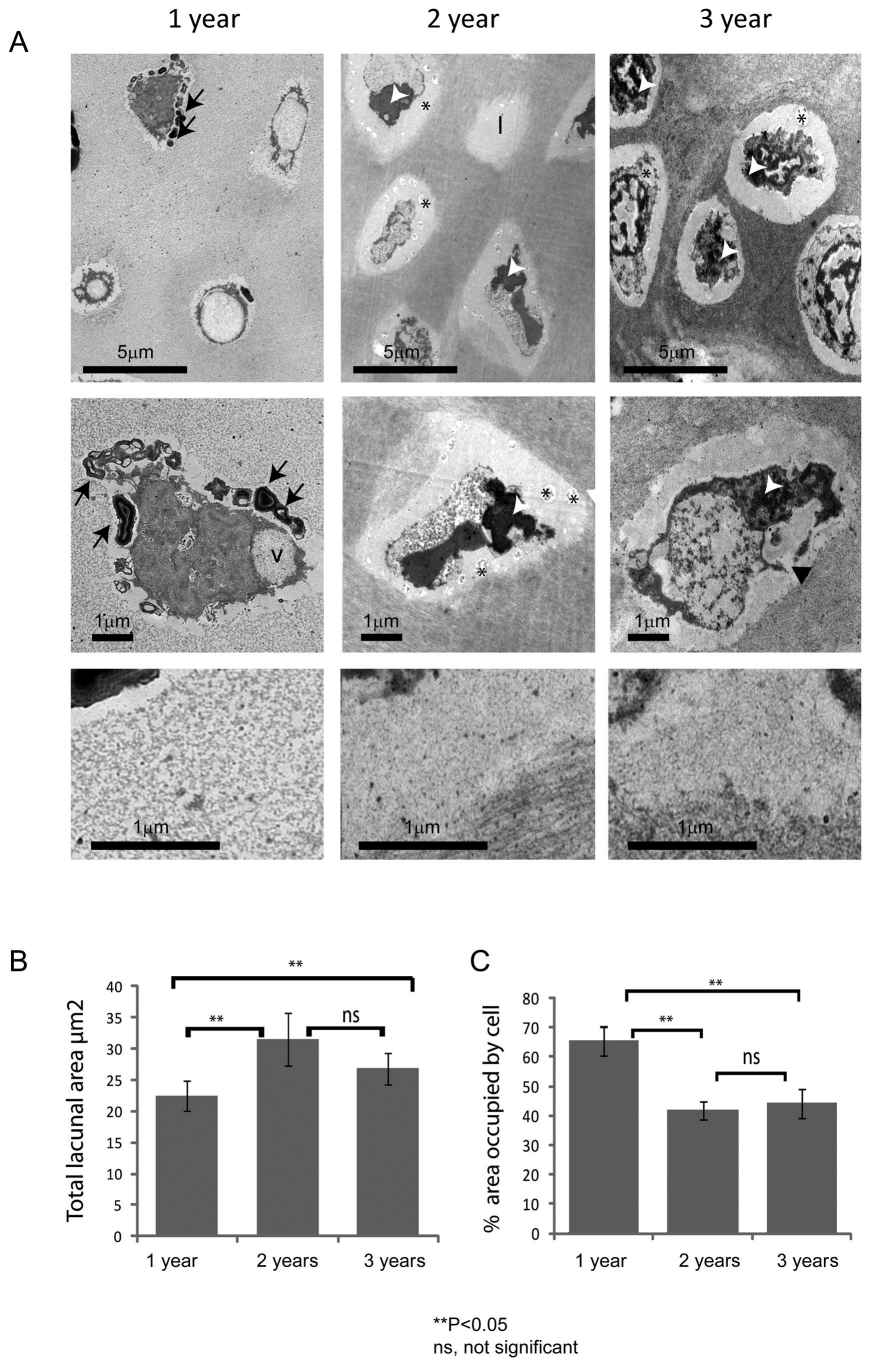
The vertebral cartilage of aged fish display changes in matrix ultrastructure and cell morphology. **A**. Representative images showing the ultrastructure of vertebral cartilage, chondrocytes and pericellular matrix (top panel, middle panel and bottom panel, respectively) at 1, 2 and 3-years (left, middle and right panels, respectively). Chondrocytes display morphologies suggestive of programmed cell death at all stages. Note prominent lacunae in 2 and 3 year samples and increase in electron density of surrounding ECM. **B**. Graph showing mean lacunal area at each age. Note significant increase in 2 and 3-year samples, relative to 1 year samples tested by One-way ANOVA; 1 vs 2 year *P*=3.26E-07, 1 vs 3 year *P*=0.00945, 2 vs 3 year *P*=0.0512. **C**. Graph showing percentage area occupied by cell at each age. Note significant decrease in 2 and 3 year samples, relative to 1 year samples tested by One-way ANOVA; 1 vs 2 year *P*=1.13E-15, 1 vs 3 year *P*=1.61E-10, 2 vs 3 year *P*=0.357. *l*, lacunae; *v*.; intracellular vacuoles; *black* *asterisks*, vesicular debris; *black* *arrows* denote myelin figures; *white* *arrows* show condensed nuclear material; *black* *arrowhead* shows discontinuity of cell membrane. Scalebar in microns.

Ultrastructural analysis of the vertebral cartilage (Figure 3A) was broadly supportive of the matrix changes seen at the light microscopic level, and also showed chronic age-related changes in cell morphology. At 1-year, cells were embedded within a relatively homogeneous ECM that had a coarse, granular appearance. At this stage, the pericellular matrix was weakly defined from the surrounding interstitial matrix. The enclosed cells were highly vacuolated and filled their lacunae; which, on occasion, contained electron-dense myelin-like figures. At 2 and 3-years, the pericellular matrix was more heterogeneous in appearance, containing both granular and fibrillar material, and was more strongly defined from the surrounding ECM. Many of the cells had highly convoluted or fragmented nuclei, and appeared retracted within conspicuously larger lacunae; some lacunae apparently devoid of cells. Image analysis showed significant differences in both the area of the lacunae (Figure 3B) and the percentage area of the lacuna occupied by the cell (Figure 3C) at 2 and 3-years, relative to 1 year samples. By 3-years, many of the cells displayed evidence of nuclear fragmentation and lysis with extrusion of cellular material into the lacuna space.

### Immunohistochemistry

**Figure 4:**
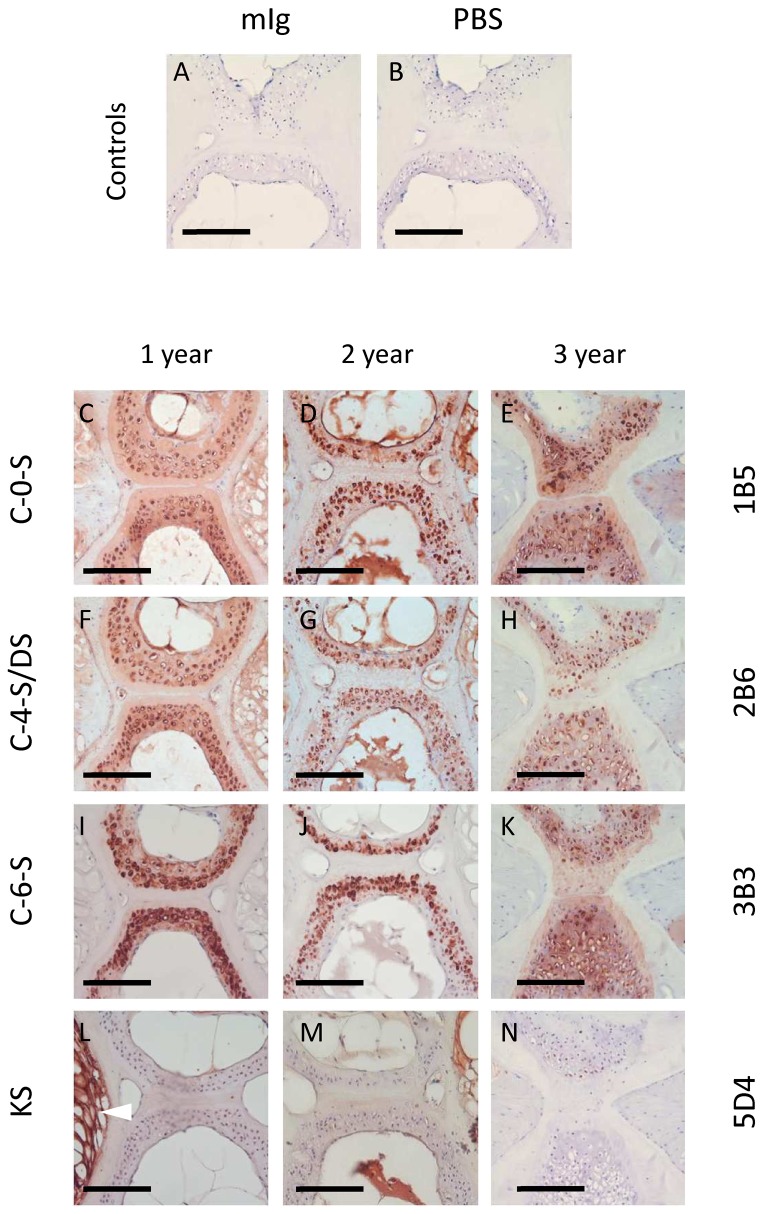
The vertebral cartilage of aging fish is rich in chondroitin, but not keratan, sulfate. **A**-**B**. Immunohistochemical labelling controls showing no non-specific binding of primary (mIg, ‘naive’ mouse immunoglobulin) or secondary antibody (PBS, phosphate buffered saline). **C**-**N**. Immunohistochemical labelling patterns of chondroitin/dermatan (C-0-S, C-4-S/DS, C-6-S) and keratan sulfate (KS) at 1, 2 and 3 years (left, middle and right panels, respectively). Note prominent pericellular labelling of CS/DS epitopes, particularly in 2 and 3 year samples. Unlike CS, KS occurs only within the notochordal tissue of the intervertebral disc (bottom left; arrowhead) and appears to diminish during aging. Scalebar represents 100 microns.

Immunohistochemical labelling controls were negative showing no non-specific antibody binding (Figure 4A, B). Immunohistochemical analysis of GAG expression patterns closely matched the Alcian blue histochemistry, with all three CS/DS isoforms examined (C-0-S, C-4-S/DS and C-6-S), but not KS, strongly localising within the pericellular micro-environment of the vertebral cartilage, with weaker labelling of the interstitial ECM (Figure 4C-K). The patterns of CS expression were broadly similar at all ages examined; however, the vertebral cartilage appeared more extensive at 3-years (Figure 4; right panel). Unlike the CS/DS isoforms, KS was specifically associated with notochordal tissue of the intervertebral disc (Figure 4 L, M). KS was highly expressed within the disc at 1 year; however, labelling became reduced with age (Figure 4L, M, N).

### Reverse Phase Ion Pair (RPIP)-high performance liquid chromatography (HPLC)

**Figure 5:**
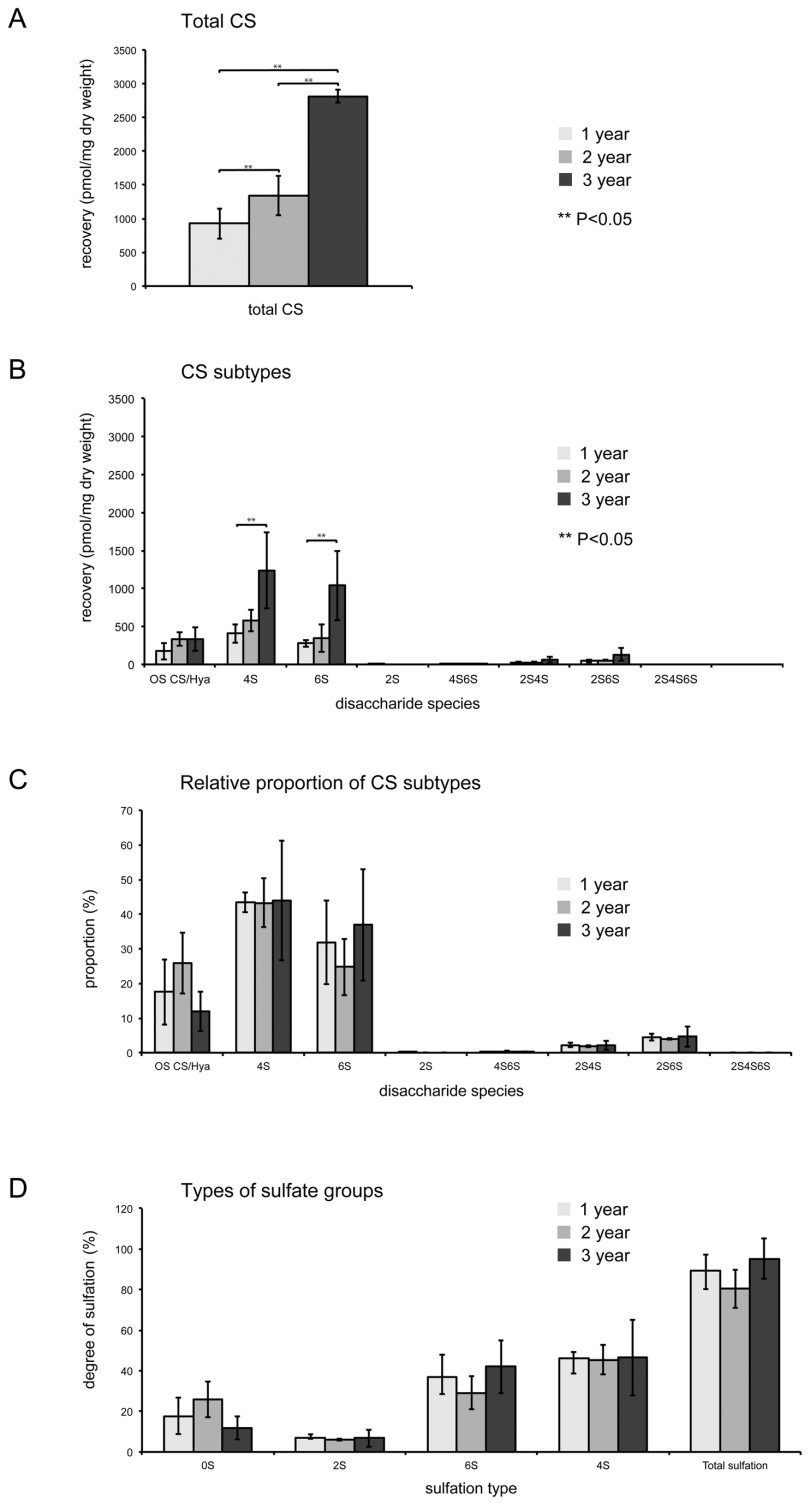
HPLC reveals increases to total vertebral chondroitin sulfate levels during aging. **A**. Total quantities of CS disaccharides recovered per mg dry weight. Note the significant increase in total CS with age tested by One-way ANOVA; 2 vs 3 year *P*=1.18E-03, 1 vs 3 year *P*=1.76E-04 **B**. Amounts of the different CS subtypes recovered. P values: for 4S 1 vs 3 year *P*=0.0486, for 6S 1 vs 3 year *P*=0.0458 C. Relative proportions of the different disaccharide species, shows no significant change to ratios of different CS subtypes. **D**. Types of sulfation observed. There is no significant change to the amount of sulfation of the disaccharides over the three ages.

Analysis of the CS content recovered from spines by RPIP-HPLC, showed a consistent age-related increase in the yield of total CS disaccharide (pmol/mg dry weight; Figure 5). Thus, at 3-years, there was approximately 3x the amount of CS disaccharide recovered per gram of dry material (2811 pmol/mg) relative to that of 1 year old spines (926 pmol/mg). However, there were no significant differences between sulfation of the different disaccharides recovered, such that the ratios between 0, 4 and 6-sulfated CS disaccharides was unchanged with increasing age. 

## Discussion

Outside of laboratory conditions, zebrafish live for approximately 3 years. With age, they show senescent phentoypes similar to those seen in higher vertebrates, such as cessation of reproductive function [29], changes to the eye (e.g. cataracts) and liver [22], tumorigenesis [30] and skeletal abnormalities including spinal curvature; ascribed previously to a loss of trunk muscle tone [23]. In this study, we describe age-related changes, with features reminiscent of osteoarthritis, specifically within the vertebral cartilage and bone of zebrafish, which may contribute to spinal deformity in this animal model.

Gross evaluation of fish showed a progressive, age-related increase in the number and severity of spinal curvatures, as recorded previously [23]. Underlying these curvatures in the most severe phenotypes, were striking radiological changes including partial or complete dislocation and abnormal vertebral morphology. Dislocations were often accompanied by focal increases in radiological bone density, suggestive of changes to bone remodelling in the vicinity; however, these were not supported by bone density data obtained by microCT. MicroCT examination showed progressive age-related abnormalities to the vertebrae, including bony outgrowths at the joint margins resembling osteophytes and focal defects and fractures within the cortical bone. Many of these features are associated with degenerative joint disease in higher vertebrates [31] and can contribute to spinal deformity and curvature. Displacement/dislocation of one vertebra relative to another is synonymous with degenerative spondylolisthesis in humans, and results from intervertebral instability or trauma [32]. Intervertebral instability also contributes to bone remodelling at joint margins and the development of vertebral osteophytes [8,9]. Osteophytosis can lead to spinal nerve compression, neuralgia and muscle wastage, manifesting in joint laxity and spinal deformity. The atrophy/sarcopenia of spinal muscles, reported previously in this organism during aging [23], may relate to the joint pathologies described here.

Examination of spinal tissue sections at both light and electron microscopic levels showed striking changes in tissue organisation and cell morphology within the vertebral cartilage with age. Collagen birefringence became less conspicuous throughout the ECM and more prominent within the pericellular micro-environment. There were accompanying changes in chondron morphology, including their enlargement and distortion, and the loss of interstitial matrix between adjacent cells, giving the tissue a disorganised appearance. The lacunae thus increased in size, whilst their cellular footprint decreased. The cells, meanwhile, displayed increasingly bizarre morphologies with ultrastructural evidence suggestive of chondroptosis [33]; which included expulsion of cellular material, cytoplasmic vacuolation, shrinkage and fragmentation. The occurrence of these age-related changes within the vertebral cartilage showed interesting parallels with some of the histopathological features of osteoarthritic cartilage in higher vertebrates [34,35,36,37,38]. Previous studies indicate that the chondron undergoes remodelling during degenerative joint disease, resulting in its distension and failure [39,40]. These changes are normally accompanied by chondrocyte proliferation and clustering, loss of matrix GAG, disorganised tissue morphology and chondrocyte cell death - both apoptosis and necrosis [33,34,36,41]. Whilst we observed evidence of programmed cell death, tissue disorganisation and remodelling, including coalescence of adjacent chondrons, interestingly there were no proliferative cell clusters, typical of osteoarthritis.

Immunohistochemistry using GAG-specific antibodies confirmed the Alcian blue staining patterns, showing that all CS/DS isoforms (i.e. C-0-S, C-4-S/DS and C-6-S), but not KS, were weakly present throughout the cartilage matrix, but concentrated specifically within the pericellular matrix. Whilst it was not apparent from the immunohistochemical labelling patterns, HPLC clearly showed a consistent age-related increase in the yield of total CS disaccharide from intact spines. Furthermore, there were no differences in sulfation between the different disaccharides recovered, thus the ratios between 0, 4 and 6 sulfated CS disaccharides remained constant with age. Similarly, there was no change in oversulfated- and 2-sulfated disaccharides with age. Degenerative joint disease is generally associated with matrix GAG depletion, an increase in the ratio of CS to KS and C-4-S to C-6-S, and an increase in native, atypical, CS sulfation epitopes [42,43,44,45,46,47,48]. It is not known whether the increase in spinal CS levels, recorded here by HPLC, result from an increase in the abundance of CS-containing PGs; an increase in GAG attachment sites or increased CS glycosylation of PG core proteins; or through increases in CS chain length [49]; however, it suggests that the spinal tissues are undergoing active tissue growth/remodelling in fish up to 3-years of age. This is supported by microCT and histological observations, which show progressive morphological changes to the vertebral tissues with age. However, it is also worthwhile noting that the changes in CS levels may relate to the associated connective tissues, e.g. ligaments and tendons, of the spine, that were included in the total CS analysis.

In adult zebrafish, CS and KS account for the majority of the total GAG content, with both being dynamically expressed during early development [50,51,52]. HS expression is high in the first 24 hours of life (~50%), but decreases as a proportion of total GAG content thereafter; whereas CS and KS increase throughout life [53]. Our study indicates that KS, identified by mAb 5D4, is largely absent from the vertebral column, only occurring within the intervertebral tissue, where it is strongly expressed by vacuolated notochordal cells in 1 year samples. Whilst KS levels were not quantified, immunohistochemical labelling suggested an age-related decrease in KS expression within the disc. This coincided with an apparent loss of vacuolated notochordal cells and their replacement with eosinophilic tissue. Notochordal cells play an important role in early spine development and their loss from the intervertebral disc with age has been proposed as a contributory factor in its degeneration [54,55,56]. The apparent loss of KS immunoreactivity from the disc is thus of interest: KS loss has been shown to be synonymous with abnormal loading of the spine, PG catabolism and predisposition towards osteoarthritis [47,57]

Overall, the age-related changes we describe within the vertebral column of zebrafish show interesting similarities with a range of pathologies normally associated with degenerative joint disease in terrestrial vertebrates [36,58]. In aqueous environments, the effects of gravity are partly offset by buoyancy; however, for propulsion, viscous drag must be overcome by muscular forces generated during swimming. These forces place significant mechanical loads on the skeleton of fish [59] and can cause spatio-temporal changes in skeletogenesis, directly affecting chondrogenesis, osteogenesis and myogenesis [17,60]. During senescence, it is conceivable that these changes may adversely affect teleost musculoskeletal tissues in a similar way to terrestrial vertebrates (i.e. through mechanical ‘wear and tear’), and lead to tissue degeneration, joint instability and spinal deformity. The sequence of events involved in the degenerative changes that lead to spinal deformity remain to be resolved; however, they may be influenced by a variety of factors - environmental, dietary and genetic - and affect a range of musculoskeletal connective tissues [12,20,23,61]. In summary, our work indicates that zebrafish, in addition to modelling skeletal development, may have validity in modelling age-related degenerative changes that affect the skeleton.
